# Quality of malaria diagnosis and molecular confirmation of *Plasmodium ovale curtisi* in a rural area of the southeastern region of Ethiopia

**DOI:** 10.1186/s12936-015-0893-y

**Published:** 2015-09-18

**Authors:** Pedro Berzosa Díaz, Patricia Mula Lozano, Jose Manuel Ramos Rincón, Luz García, Francisco Reyes, Agustín Benito Llanes

**Affiliations:** National Centre of Tropical Medicine, Institute of Health Carlos III, C/Monforte de Lemos 5, 28029 Madrid, Spain; Department of Internal Medicine, Hospital General Universitario de Alicante, Alicante, Spain; Gambo General Rural Hospital, Gambo, West Arsi Province, Oromia Region Ethiopia

**Keywords:** Malaria, *Plasmodium*, Microscopy diagnoses, *P. ovale*, PCR diagnoses, Sequencing

## Abstract

**Background:**

Approximately 50 million people (60 %) live in malaria risk areas in Ethiopia, at altitudes below 2000 m. According to official data, 60–70 % of malaria cases are due to *Plasmodium falciparum*, and 40–30 % by *Plasmodium vivax.* The species *Plasmodium ovale* was detected in 2013 in the northwest of the country, being the first report of the presence of this species in Ethiopia since the 60 s. The aim of this study was to assess the diagnosis by microscopy and PCR, and demonstrate the presence of other *Plasmodium* species in the country.

**Methods:**

The survey was conducted in Bulbula, situated in the Rift Valley (West Arsi Province, Oromia Region). From December 2010 to October 2011, 3060 samples were collected from patients with symptoms of malaria; the diagnosis of malaria was done by microscopy and confirmation by PCR.

**Results:**

736 samples were positive for malaria by microscopy. After removing the 260 samples (109 positives and 151 negatives) for which it was not possible to do PCR, there were a total of 2800 samples, 1209 are used for its confirmation by PCR and quality control (627 are positives and 582 negatives by microscopy). From the 627 positive samples, 604 were confirmed as positive by PCR, 23 false positives were detected, and the group of 582 negative samples, 184 were positive by PCR (false negatives), which added to the previous positive samples is a total of 788, positive samples for some species of *Plasmodium* sp. 13.3 % more positives were detected with the PCR than the microscopy. Importantly, 23 samples were detected by PCR as *P. ovale*, after the sequencing of these samples was determined as *P. ovale curtisi*.

**Conclusions:**

The PCR detected more positive samples than the microscopy; in addition, *P. ovale* and *P. ovale/P. vivax* were detected that had not been detected by microscopy, which can affect in the infection control.

## Background

Over 60 % of the total Ethiopian population (50–84.2 million) lives in areas at risk of malaria, generally at elevations below 2000 m above sea level. In 2014, the Federal Ministry of Health Ethiopia (FMOH) Global Fund Concept Note stratified Ethiopian malaria transmission risk as follows within 835 districts by population (%) and by annual parasite incidence per thousand (API): High [>100/1000, 11 million (13 %)]; Medium [5–99.9/1000; 28.1 million (34 %)], Low [0.1–4.9, 11.1 million (13 %)], and Malaria-Free [~0, 33.6 million (40 %)]. According to the Federal Ministry of Health Ethiopia (FMOH), in 2011/2012, malaria was the leading cause of outpatient visits, accounting for 17 % of all outpatient visits, and 8 % of health facility admissions among all age groups. Malaria is one of the top ten causes of inpatient deaths among children aged less than 5 years and in older individuals according to the Health Management Information System (HMIS) data. In 2012/2013, there were 57,503 public sector malaria hospitalizations, 4,984,266 malaria outpatient cases, and 2,942,031 laboratory-confirmed *Plasmodium falciparum* outpatient malaria cases, and 1,258,131 *Plasmodium vivax* cases according to the annual micro-plan [1].

According to official resources the major *Plasmodium* species causing malaria in Ethiopia are *P. falciparum* (about 60 % of cases) and *P. vivax* (about 40 % of cases), with the former being the cause of the most severe clinical manifestations and most deaths [2–4]. Recent therapeutic efficacy trials have proven that artemisinin and chloroquine have adequate effectiveness for treating these pathogens, respectively. To date, major problems with emerging drug resistance and counterfeit or substandard anti-malarial drugs have not yet been detected in Ethiopia [1]. Control measures established in the area where the samples have been collected were established by the Ministry of Health of Ethiopia: microscopic diagnosis, treatment with artemisinin-based combination therapy (ACT), protection with bed nets impregnated with insecticide and indoor insecticide spraying.

In general, light microscopy examination of blood smears is still considered the “gold standard” for laboratory diagnosis of malaria [5]. In many malaria endemic regions, however, microscopic diagnosis displays certain limitations, such as a shortage of skilled microscopists, inadequate quality control [5, 6] and the possibility of misdiagnosis due either to low parasitaemia or to mixed infections [5, 7, 8]. Moreover, in Ethiopia, as in other endemic areas, this method of diagnosis is unavailable to the most distant health facilities, for which clinical diagnosis is still widely used, leading to cross-diagnosis with other diseases [9, 10].

Sometimes it is difficult to determine the species of *Plasmodium* that is detected in the patient by microscopic diagnosis, maybe for lack of training of the microscopists, with the consequence that some species are not reported in the country, as in the case of *Plasmodium ovale*. This species has a similar morphology to *P. vivax*, and the two may be confused by microscopy. Although this does not have implications in the treatment, because the patient will receive the same treatment as with *P. vivax*, it has important implications in the malaria epidemiology and in the malaria parasite distribution. Despite the first report of *P. ovale* in Ethiopia in 1969 [11], the knowledge of the distribution and epidemiology of different human malaria species, particularly *P. ovale* spp., and *Plasmodium malariae* [12] is rather limited in the entire country. Alemu et al. [13] detected the presence of *P. ovale* in 2013 in Bahir Dar, a town on Tana Lake, by PCR and sequencing—a smilar approach as used in the present study.

At present, diagnosis of malaria in reference laboratories in non-endemic areas is performed with the aid of molecular biology techniques, such as PCR. PCR has been confirmed as having a lower detection threshold of 0.7–0.02 parasites/μl, compared with real time PCR [14, 15].

The aim of this study was to assess the presence of other malaria species, and the capacity of the microscopists to detect and identify *Plasmodium* species. This study has used the semi-nested multiplex PCR [16] to evaluate the sensitivity and specificity of optical microscopy for diagnosis of the *Plasmodium* infection among all patients suspected of having malaria in a health centre of a rural area of southeastern Ethiopia

## Methods

### Survey site, sample collection and microscopy examination

**Fig. 1:**
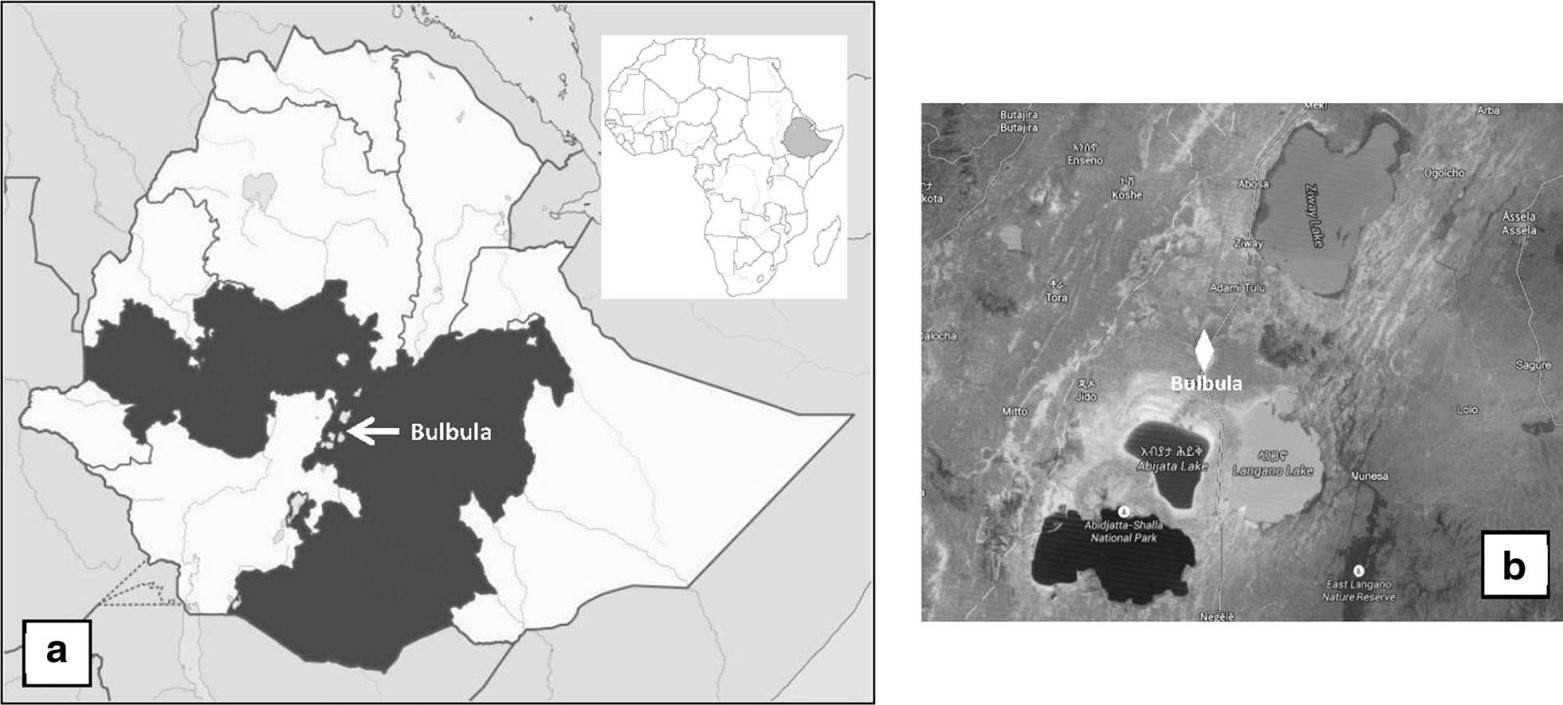
Map of the study area. **a** Ethiopia map where appears the Oromia Region, and the location of Bulbula, rural area where the study was carried out. **b** Satellite map where appears located Bulbula and lakes that surround it, Ziway Lake, Langano Lake and Abijata Lake, a fact that gives it a peculiar epidemiology of malaria. Images extracted from http://www.es.wikipedia.org/wiki/Orom%C3%ADa and https://www.google.es/maps/

The survey was conducted in Bulbula, a town located in West Arsi Province, Oromia Region (Fig. 1). All patients who attended the Bulbula Health Centre, with fever and suspected of having uncomplicated malaria, were included in this study. A total of 3060 samples were collected from suspected malaria patients from December 2010 to October 2011, with two capillary blood samples, one for microscopic diagnosis, and a filter paper (Whatman© 3MM) for diagnostic confirmation by PCR. The blood on the filter paper was air dried, then stored in double zip-lock plastic bags with silica gel at 4 °C, and subsequently transported to the National Centre for Tropical Medicine, Institute of Health Carlos III, Madrid (Spain) for diagnostic confirmation by PCR. All patients signed informed consent forms and the study received ethical clearance.

Thin films were stained with 5 % Giemsa solution for 20 min, and examined by microscopists from the Health Centre of Bulbula. The normal protocol for microscopic diagnosis was followed, each slide was assessed by two microscopists, and, if there was a discrepancy, a third microscopist assessed the slide. Parasite density was not evaluated because the routine work of health centres in rural areas of Ethiopia involves checking for the presence or absence of parasites but does not extend to calculating parasite densities.

### Ethics

Ethical clearance was obtained from the Rural General Hospital of Gambo (West Arsi Province, Oromia Region), a hospital which depends on the Health Centre of Bulbula. Informed consents forms were obtained from each patient or a parental/legal guardian. Participation in the study was voluntary and had no influence on the treatment at the health centre. All data is confidential.

### PCR analysis and DNA extraction

In line with quality assurance programmes (WHO 2005), all positive samples by microscopy (736 samples) and 10 % of negative microscopy samples were analysed by PCR. Since some of the negative microscopy samples tested positive with PCR, an additional 10 % was examined, so that a total of 582 negative samples were analysed by PCR, as quality control. 109 samples were excluded, which proved to be positive, and 151 negatives samples by microscopic diagnosis, owing to the lack of filter paper and problems with the numbering; this could be considered a limitation of the study by PCR. DNA extraction was performed on the filter paper samples using commercial kits (Speedtools tissue DNA Extraction Kit, Biotools, Spain). The PCR diagnosis is a Semi-nested Multiplex PCR, and it was performed, as described by the author [16]. The PCR product was subjected to ultraviolet transilluminator using electrophoresis on 2 % agarose gels stained with ethidium bromide.

### Sequence analysis of *Plasmodium ovale*

23 samples diagnosed for *P. ovale* by PCR were sequenced for specific characterization, as *P. ovale wallikeri* or *P. ovale curtisi*. This was done throughout the partial sequencing of the small subunit of ribosomal RNA (ssrRNA) gene, with the primers New PLF Sh/New Rev Sh [17]. The amplified products were purified with the QI Aquick PCR Purification Kit (WERFEN, Spain SAU) and sequenced with the big Dye Terminator v3.1 Cycle Sequencing in an ABI PRISM 3730 Analyser. It is necessary for sequencing to have 20 ng of the amplification fragment and 5 mM of each primer (forward and reverse). All sequences were analysed by free web pages [18, 19], and subsequently were compared in GeneBank for knowing their identities.

### Data-analysis

It was carried out a descriptive analysis and obtained prevalence estimates and their 95 % confidence intervals (CIs) through microscopy, PCR diagnoses and sequences results (McCallum Layton, Stats Calculator). Sensitivity and specificity were calculated with the Software Epidat 3.1.

## Results

A total of 3060 samples of patients suspected of malaria were collected in Bulbula Health Centre, from them 1553 females and 1507 males, age range 1 month to 80 years (mean = 13.4 years; median = 10 years).

### Microscopy

From a total of 3060 individuals, 736 (24 %; CI 24.49–25.51 %) were diagnosed as *Plasmodium* sp. and 2324 as negatives (76 %) for infection by *Plasmodium* sp. From the species detected by microscopy were: 483 samples as *P. vivax* (16 %; 95 % CI 14.7–17.38.96 %), 243 as *P. falciparum* (8 %; 95 % CI 7.04–8.96 %), and 10 as mixed infections *P. falciparum/P. vivax* (0.3 %; 95 % CI 0.11–0.49 %) (Table 1).

**Table 1:** Microscopy results

Species	N	%	95 % CI	
*Plasmodium* sp.	736	24	24.49	25.51
*P. falciparum*	243	8	7.04	8.96
*P. vivax*	483	16	14.7	17.3
*P. falciparum* + *P. vivax*	10	0.3	0.11	0.49
Negatives	2324	76	74.49	77.51
Total	3060	100		

The table shows the frequency of the different species of *Plasmodium* sp. and negative samples diagnosed by microscopy

### PCR

**Fig. 2:**
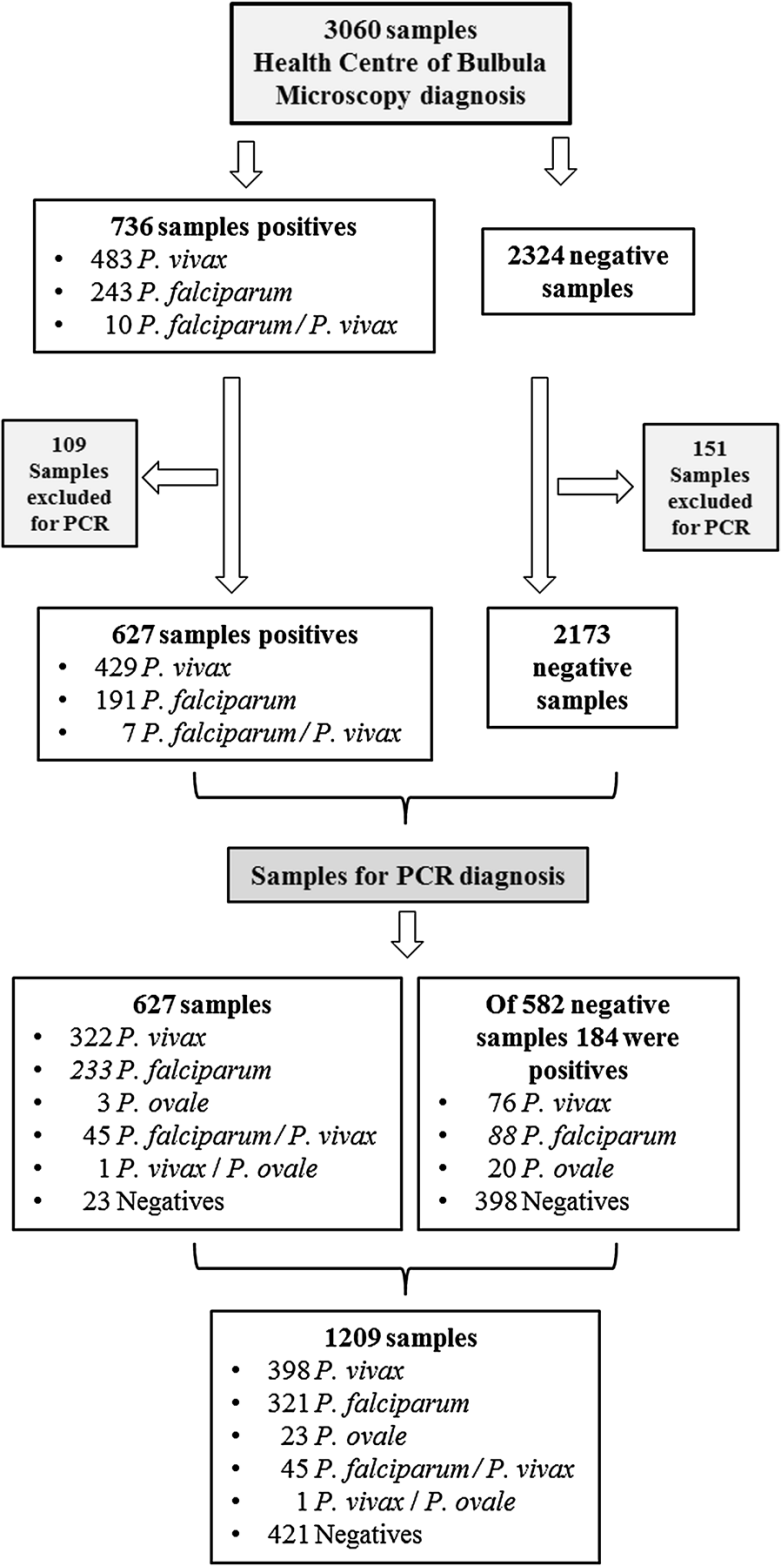
Flowchart of sample selection process. The figure shows the number of positive samples and negatives diagnosed by microscopy, the number of samples no valid for PCR and finally the number of samples diagnosed by this technique

As shown in Table 2, the 736 positive samples by microscopy were taken for diagnosis by PCR, but 109 samples were excluded due to a lack of filter paper at sampling time, an error in numbering or a small amount of blood on the filter paper. 627 positive samples were analysed (429 *P. vivax*, 191 *P. falciparum*, seven *P. falciparum/P. vivax* mixed infections) for the confirmation of species by PCR. The process of selection of samples for the diagnosis by PCR and its results appear in Fig. 2.

**Table 2:** Reasons for exclusion of the samples

	Total positives by microscopy	Absence filter paper	Error numbering	Small amount of blood on filter paper	Total eliminated	Total for diagnosis by PCR
*P. vivax*	483	9	43	2	54	429
*P. falciparum*	243	6	41	5	52	191
*P. falciparum* + *P. vivax*	10	0	1	2	3	7
Total negatives	2324	6	40	108	151	2173
Total	736	15	85	9	109	627

The table shows the reasons for excluding filter papers for its diagnosis by PCR in each of the species and in the negative group of samples diagnosed by microscopy

Regarding the negative samples, it was necessary to confirm those diagnosed by microscopy. 10 % of the samples (306 samples) were selected and were used as control microscopy-negative samples. Since a proportion of this 10 % proved to be positive, a further 10 % of the negative samples (276 samples) were analysed. Moreover, of the total samples shown to be negative by microscopy (2324), 151 had to be excluded for the same reasons as before.

**Fig. 3:**
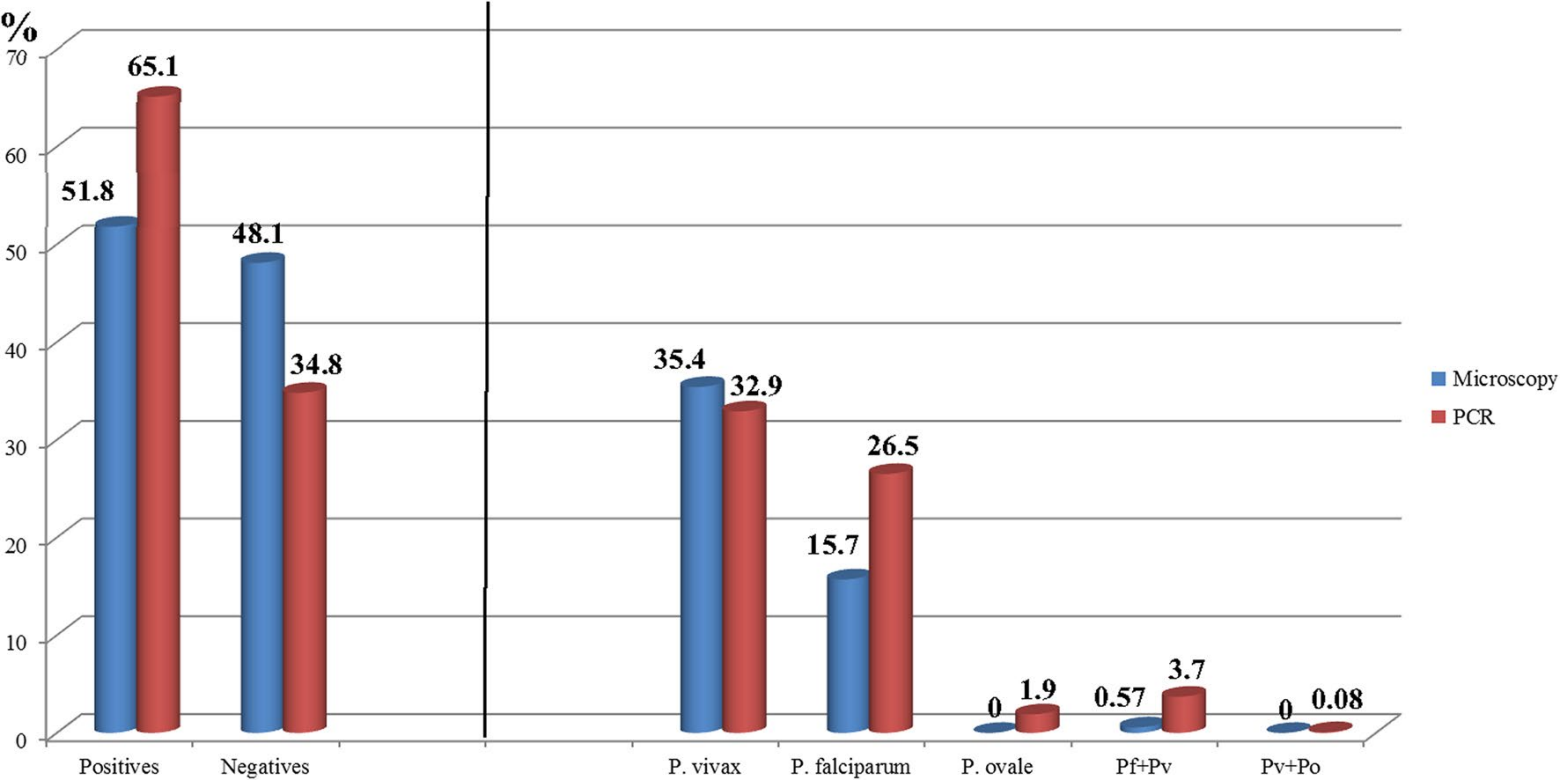
Comparison of the diagnosis by microscopy and PCR. In the first part of the figure appears the comparison between the frequencies of positive and negative samples diagnosed by microscopy and by PCR. In the second part of the figure shown the comparison between the frequencies of each species diagnosed by microscopy and PCR

After the study of the selected 1209 samples analysed by PCR (see Table 3), 321 samples were detected as *P. falciparum* (26.5 %; 95 % CI 24.01–28.99 %), 398 *P. vivax* (33 %; 95 % CI 30.35–35.65 %), 23 *P. ovale* (2 %; 95 % CI 1.21–2.79 %), 45 *P. falciparum/P. vivax* (4 %; 95 % CI 2.9–5.1 %) and one *P. vivax/P. ovale* (0.08 %; 95 % CI −0.08 to 0.24 %) and 421 (35 %; 95 % CI 32.31–37.69 %) negatives samples. In the group of 627 positive samples by microscopy, 23 negative samples were confirmed negative by PCR (1.9 %) (i.e. false positives). From the total of 582 negative samples analysed, 398 samples were negative by microscopy (68.4 %; 95 % CI 64.62–72.18 %). The rest of the 184 samples were positive for one species (false negatives, 15.2 %), 88 for *P. falciparum* (15 %; 95 % CI 12.1–17.9 %), 76 for *P. vivax* (13 %; 95 % CI 10.27–15.73 %) and 20 for *P. ovale* (3.4 %; 95 % CI 1.93–4.87 %). To summarize these results: after removing the 260 samples (109 positive and 151 negative) of which it was not possible to do the diagnostic PCR, we had a total of 2800 samples, of which 1209 are used for its confirmation by PCR and quality control [627 were positives by microscopy (22.4 %) for* Plasmodium* sp. and 582 negatives (77.6 %)]. From the 627 positive samples diagnosed by microscopy, 604 were confirmed as positive by PCR, therefore 23 false positives were detected (see Table 3), and the group of 582 negative samples selected for quality control of the diagnosis by microscopy, 184 were positive by PCR (false negatives, 15.2 %), which added to the previous positive samples is a total of 788 positive samples (28.1 %) for some species of* Plasmodium* sp. It was detected 13.3 % more positives with the PCR than the microscopy (see Fig. 3).

**Table 3:** Confirmation and/or correction of the microscopic diagnosis by PCR

Species by PCR	627 samples detected by microscopy									Confirmation of the negative samples			Total	%	95 %CI	P value
	*P. falciparum* by microscopy N = 191			*P. vivax* by microscopy N = 429			*P. falciparum* + *P. vivax* by microscopy N = 7			N = 582						
	N	%	95 % CI	N	%	95 % CI	N	%	95 % CI	N	%	95 % CI				
*Pf*	183	95.8	92.96/98.64	48	11.2	8.2214.18	2	28.6	−4.88/62.08	88	15	12.1/17.9	321	26.5	24.01/28.99	0.000
*Pv*	2	1.05	−0.4/2.5	320	74.6	70.48/78.72	0	0	0	76	13	10.27/15.73	398	33	30.35/35.65	0.063
*Po*	1	0.5	−0.5/1.5	2	0.5	−0.17/1.17	0	0	0	20	3.4	1.93/4.87	23	2	1.21/2.79	–
*Pf* + *Pv*	2	1.05	−0.4/2.5	38	8.8	6.12/11.48	5	71.4	37.92/104.88	0	0	0	45	3.7	2.64/4.76	0.000
*Pv* + *Po*	0	0	0	1	0.23	−0.22/0.68	0	0	0	0	0	0	1	0.08	−0.08/0.24	–
Negatives	3	1.6	−0.18/3.38	20	4.7	2.7/6.7	0	0	0	398	68.4	64.62/72.18	421	35	32.31/37.69	0.000
Total	191			429			7			582			1209			

The columns show the distribution of each of the species diagnosed by microscopy, and the rows show the distribution of species after the diagnosis by PCR

This biggest frequency in the diagnosis by PCR, may be due to that 184 samples diagnosed as negatives by microscopy were positive (false negatives) after the diagnosis by PCR, as shown in Table 3. 88 samples from these 184 false negatives, 48 %, were finally diagnosed as *P. falciparum* by PCR. This failure in the diagnosis by microscopy could be due to the difficulty of detecting parasites by microscopy when in very low density. On the other hand, the new data of the frequency of *P. falciparum* by PCR fits better with the prevalence official data of the country (60 %). After the calculation of specificity and sensitivity, PCR as gold standard, the sensitivity was 76.65 % (95 % CI 73.63–79.67) and specificity of 94.54 (95 % CI 92.25–96.83), see Table 4.

**Table 4:** Sensitivity and specificity estimates with 95 percent CI, comparison of the diagnosis, PCR as a gold standard

Microscopy	Reference test-PCR		
	Positives	Negatives	Total
Positives	604	23	627
Negatives	184	398	582
Total	788	421	1209

	Value	95 % CI	
Sensitivity (%)	76.65	73.63–79.67	
Specificity (%)	94.54	92.25–96.83	
Predictive value+ (%)	96.33	94.78–97.88	
Predictive value− (%)	68.38	64.52–72.25	

### Sequencing analysis of *Plasmodium ovale* detected by PCR

The small subunit of ribosomal RNA (rRNA) partial gene was amplified and sequenced from the 23 *P. ovale* samples detected by PCR. After sequence analysis and comparison with GeneBank, 23 samples detected as *P. ovale* provded to be *P. ovale curtisi* after the sequencing. *Plasmodium ovale wallikeri* was not detected in the group of samples; this does not mean it does not exist in this area of study.

## Discussion

This study shows, that in the context of a rural area in Ethiopia, the microscopy has low overall sensitivity and specificity for the diagnosis of *Plasmodium* species, in fact a new species, *Plasmodium ovale* spp., has been detected in this area.

It is important to emphasize the high number of false negatives (184) and false positives (23) detected and the impact this has on infection control, as they are patients who have not been treated, or they received treatment without needing it, and this could facilitate transmission. This highlights the difficulty of detecting parasites by microscopy when it appears a low parasite density. An improvement in microscopic diagnosis would prevent both over-treatments of patients and over-exposure to anti-malarial drugs. This would help to reducte costs, side effects, and most importantly, the risk of emergence and spread of drug resistance [20–22]. Furthermore, the diagnostic of *P. falciparum* by microscopy, in both mono-infection and mixed infections, is low with respect to that observed for *P. vivax* by microscopy. This result is quite interesting, in view of the fact that in Ethiopia *P. falciparum* is considered the main species, with a prevalence of around 60 %, according to official data.

On the other hand, the 38 patients who were diagnosed as *P. vivax* (mono-infection) by microscopy, but who were actually co-infected with *P. falciparum*–*P. vivax*, detected by PCR, were treated with chloroquine (CQ), and so they received the wrong treatment.

*Plasmodium vivax*-infected patients are only treated with CQ in accordance with Ethiopian treatment guidelines [9]. The use of CQ with primaquine, a tissue schizonticide, would eliminate latent hypnozoites of *P. vivax* in the liver and consequently reduce the possibility of recurrences [23]. However, primaquine is not used in this area, because the risk of re-infection is very high. Clinicians point out that the damage that the primaquine may produce is worse than the infection itself.

23 cases of *P. ovale* were detected by PCR, after the sequencing of the small subunit of ribosomal RNA (ssRNA) gene of these samples; they were confirmed as *P. ovale curtisi*. However, *P. ovale wallikeri* was not detected. This is an important epidemiological fact, because for the first time, a new species of *Plasmodium* has been detected in this area of Ethiopia. The last time that *P. ovale* was reported in Ethiopia was in 1969 [11], but it was not until 2013 [13] when the presence of *P. ovale curtisi* and *P. ovale wallikeri* was published in the northwest of Ethiopia. This is the first time *P. ovale curtisi* has been detected in southeastern Ethiopia, in Bulbula, a village in the Rift Valley. It is an area with a high malaria transmission.

Due to the unavailability of molecular techniques in these areas, a good microscopy confirmation method would be the rapid diagnostic test of malaria. Despite the fact that it does not match the PCR sensitivity, it would nevertheless reinforce the results obtained by microscopic diagnosis [20, 24–26]. The introduction of this test must be accompanied by adequate training [27, 28]. Taking into account the results obtained in the study, it would be desirable to provide training and updated courses on malaria to improve the microscopic identification of *Plasmodium* species in rural health centres, where scarcity of resources is greater than in other places.

Although Ethiopia has some reference laboratories for infectious diseases in different regions, a major challenge facing the country is to establish more direct contacts between rural areas and these reference laboratories, where molecular techniques could be standardized and thus to support the microscopic diagnosis in rural areas, in addition to perform a quality control of diagnosis methods. These reference laboratories could perform prevalence and drug-resistance studies; as is done in some regions of Ethiopia [29]. It would be necessary to extend this kind of studies to all regions, which would allow to obtain more reliable epidemiological data.

## Conclusions

The analysis by PCR of the samples collected in Bulbula (south-eastern Ethiopia), has detected more positives samples for malaria than the diagnosis by microscopy made in this health centre, which could have serious effects on infection control. Throughout the diagnosis by PCR and subsequent sequencing, *P. ovale curtisi* was detected for the first time in this area. In view of the results obtained in diagnostic microscopy, it would be good to provide training and update courses for malaria diagnosis regularly, to improve the quality of diagnosis. In addition, it would also be advisable for these health centres to have another confirmation technique, such as rapid diagnostic tests for malaria.
